# Assessing the Effectiveness of Direct Data Merging Strategy in Long-Term and Large-Scale Pharmacometabonomics

**DOI:** 10.3389/fphar.2019.00127

**Published:** 2019-02-20

**Authors:** Xuejiao Cui, Qingxia Yang, Bo Li, Jing Tang, Xiaoyu Zhang, Shuang Li, Fengcheng Li, Jie Hu, Yan Lou, Yunqing Qiu, Weiwei Xue, Feng Zhu

**Affiliations:** ^1^College of Pharmaceutical Sciences, Zhejiang University, Hangzhou, China; ^2^School of Pharmaceutical Sciences and Collaborative Innovation Center for Brain Science, Chongqing University, Chongqing, China; ^3^School of International Studies, Zhejiang University, Hangzhou, China; ^4^Zhejiang Provincial Key Laboratory for Drug Clinical Research and Evaluation, The First Affiliated Hospital, Zhejiang University, Hangzhou, China

**Keywords:** direct data merging, classification capacity, robustness, false discovery rate, long-term and large-scale metabolomics

## Abstract

Because of the extended period of clinic data collection and huge size of analyzed samples, the long-term and large-scale pharmacometabonomics profiling is frequently encountered in the discovery of drug/target and the guidance of personalized medicine. So far, integration of the results (*ReIn*) from multiple experiments in a large-scale metabolomic profiling has become a widely used strategy for enhancing the reliability and robustness of analytical results, and the strategy of direct data merging (*DiMe*) among experiments is also proposed to increase statistical power, reduce experimental bias, enhance reproducibility and improve overall biological understanding. However, compared with the *ReIn*, the *DiMe* has not yet been widely adopted in current metabolomics studies, due to the difficulty in removing unwanted variations and the inexistence of prior knowledges on the performance of the available merging methods. It is therefore urgently needed to clarify whether *DiMe* can enhance the performance of metabolic profiling or not. Herein, the performance of *DiMe* on 4 pairs of benchmark datasets was comprehensively assessed by multiple criteria *(classification capacity, robustness and false discovery rate). As* a result, integration/merging-based strategies (*ReIn* and *DiMe*) were found to perform better under all criteria than those strategies based on single experiment. Moreover, *DiMe* was discovered to outperform *ReIn* in *classification capacity* and *robustness*, while the *ReIn* showed superior capacity in controlling *false discovery rate*. In conclusion, these findings provided valuable guidance to the selection of suitable analytical strategy for current metabolomics.

## Introduction

Liquid chromatography-mass spectrometry has been widely applied in pharmaceutical and clinical metabolomics to comprehensively reveal metabolic alteration in given biological system (Paglia and Astarita, 2017; Fu et al., 2018; Tang et al., 2018; Yang et al., 2019), identify biomarkers and therapeutic targets for a variety of complex diseases (Zhu et al., 2009; Yang et al., 2016; Hu et al., 2017; Li et al., 2017c, 2019) and illuminate mechanism of action of drugs or drug candidates (Chen et al., 2017; Li X. et al., 2018; Li X.X. et al., 2018; Xue et al., 2018b; Zhang et al., 2018). Because of the extended period of clinical data collection and huge size of analyzed samples, the long-term and large-scale metabolomic profiling is frequently encountered in current medical study to identify physiological perturbation in various living systems (Zhao et al., 2016; Zheng et al., 2018), analyze time-dependency of metabolic alteration (He et al., 2015; Han et al., 2018) and evaluate therapy and patient stratification in personalized medicine (Li et al., 2017a; Wang et al., 2017a). Data from large-scale metabolomics are generally collected over long period varying from months to years and must be divided into batches, which requires a comprehensive consideration of all data of various batches or studies (Brunius et al., 2016; Li Y.H. et al., 2018). So far, *ReIn* of multiple experiments in large-scale metabolomics has been applied to enhance the reliability and robustness in cancer-related metabolites profiling (Goveia et al., 2016; Xue et al., 2018a) and marker discovery for prediabetes or diabetes patients (Guasch-Ferre et al., 2016; Wang et al., 2017b).

**FIGURE 1:**
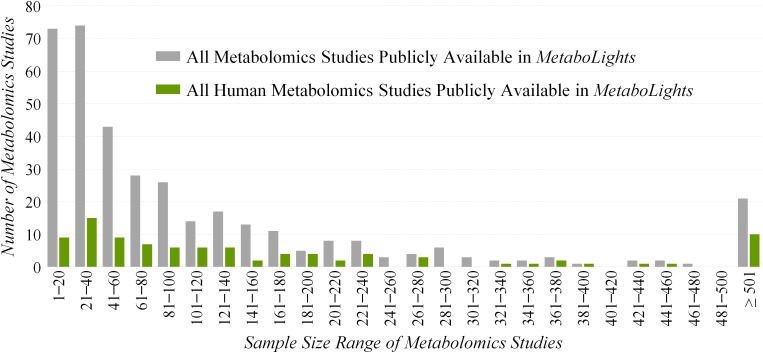
Distribution of the sample sizes of all (gray) and human (green) metabolomics studies publicly available in the *Metabolights* database.

However, *ReIn* precludes the reanalysis of original data due to the lack of quantitative metabolomics data and inevitably results in inadequate statistical power (Goveia et al., 2016). Due to the necessity of quantitative data, a database named *MetaboLights* providing such information has been established (Kale et al., 2016), which makes the reanalysis or integrated analysis of the quantitative data possible and convenient (Haug et al., 2013). Based on our comprehensive investigation on all metabolomics studies in *MetaboLights* (Figure 1), the sample sizes of the majority (>65%) and almost half (>45%) of these studies are less than 100 and 50, respectively. As reported, a total cohort of over 100 samples is essential for the identification of a maximum of statistically significant variations in any metabolic exploration (Billoir et al., 2015). Since the bias of current metabolic explorations is reported to come frequently from the inadequacy of studied samples (Zhang et al., 2006; Subramanian, 2016), there is an urgent need to maximally enlarge the sample size and in turn enhance the statistical power of a given metabolomics study (Button et al., 2013).

Till now, *DiMe* strategy has been adopted in OMIC studies which effectively enlarges the size of studied samples (Lazar et al., 2013; Li et al., 2014; Switnicki et al., 2016). In particular, new breast cancer biomarkers are identified by combining RNA-seq gene expression data (Switnicki et al., 2016); novel alternative splicing is found by collectively analyzing multiple RNA-seq datasets (Li et al., 2014); the removal of batch effects from transcriptomics data is investigated by microarray data integration (Lazar et al., 2013). Due to the enlargement of studied samples, *DiMe* demonstrates potential enhancements in the accuracy, consistency and robustness of OMIC data analysis (Larsson et al., 2006; Goveia et al., 2016), and is proposed to significantly increase statistical power, reduce experimental bias, enhance reproducibility and improve overall biological understanding (Zhao et al., 2016). However, compared with *ReIn*, the *DiMe* of multiple experiments has not yet been widely used in current metabolomics studies, which may be attributed to two major factors (Zhao et al., 2016; Li et al., 2017b). The first is the difficulty in removing the unwanted variations among experiments and inexistence of prior knowledges on the performance of the available merging methods (Zhao et al., 2016). In other word, it is still elusive whether the *DiMe* can effectively enhance the performance of metabolic profiling (Soto-Iglesias et al., 2016). The second is the existence of multiple criteria to assess the performance of *DiMe* and the great difficulty of selecting the optimal one (Li et al., 2017b; Valikangas et al., 2018). As reported, a multiple criteria evaluation is more effective than the single one in assessing the reliability of integration (Lee and Smith, 2012), and a collective consideration of multiple criteria is therefore recommended to thoroughly evaluate the applied strategy from different perspectives (Li et al., 2017b; Valikangas et al., 2018). All in all, because of the distinct underlying theory of these criteria, it is very essential to systematically assess the performance of *DiMe* strategy by collectively considering all criteria.

In the study, comprehensive evaluation of different analytical strategies was conducted by assessing their *classification capacity, robustness* and *false discovery rate*. First, based on a systematic review of *MetaboLights* a number of benchmark studies were identified to accomplish this assessment. Then, the integration/merging-based strategies (*ReIn* and *DiMe*) together with the strategies based on single experiment were collectively evaluated by multiple criteria. In conclusion, these findings provided a valuable guidance to the selection of suitable analytical strategy in a given metabolomics study.

## Materials and Methods

### Collection of Metabolomics Datasets to Assess the Performance of *DiMe* Strategy

A systematic search in the *MetaboLights* database (Haug et al., 2013) was collectively conducted to discover benchmark datasets for the performance assessment of *DiMe*. First, the *MetaboLights* was searched by the keyword “mass spectrometry,” which resulted in 339 projects (September 16, 2018). Second, several criteria were used to ensure the availability and processability of raw metabolomics data, which included (a) complete set of raw data files, (b) well-defined parameters (mz value, range of retention time), (c) enough samples (>10) in each experiment, (d) same classes of both cases and controls in different experiments, and (e) clear description on the sample groups. The application of the above criteria to those 339 projects resulted in eight benchmark metabolomics datasets of varied sample sizes. In particular, these eight datasets included (1) a UPLC-QTOF MS dataset based on the serums of 59 patients of HCC and 129 CIR patients collected at Georgetown University Hospital (GUH) and run in positive mode from an experiment conducted in May 2010 (Xiao et al., 2012), (2) a metabolomics benchmark dataset of the MS positive mode based on the serums of 13 HCC and 50 CIR patients collected at GUH in July 2010 (Xiao et al., 2012), (3) a UPLC-QTOF MS dataset based on the serums of 59 HCC and 129 CIR patients collected at GUH and run in negative mode from an experiment conducted in May 2010 (Xiao et al., 2012), (4) the benchmark dataset of MS negative mode based on the serums of 13 HCC and 50 CIR patients collected at GUH in July 2010 (Xiao et al., 2012), (5) the UPLC-QTOF MS dataset based on the serums of 20 HCC and 25 CIR patients collected from Egypt and run in positive mode (Xiao et al., 2012), (6) the metabolomics benchmark dataset of the MS positive mode based on the serums of 20 HCC and 24 CIR patients collected in Egypt (Xiao et al., 2012), (7) UPLC-QTOF MS dataset based on the serums of 20 HCC and 25 CIR patients collected in Egypt and run in negative mode (Xiao et al., 2012), and (8) the benchmark dataset of MS negative mode based on the serums of 20 HCC and 24 CIR patients collected from Egypt (Xiao et al., 2012).

### Direct Data Merge (*DiMe*) Strategy Used in This Study Based on the m/z Values

**FIGURE 2:**
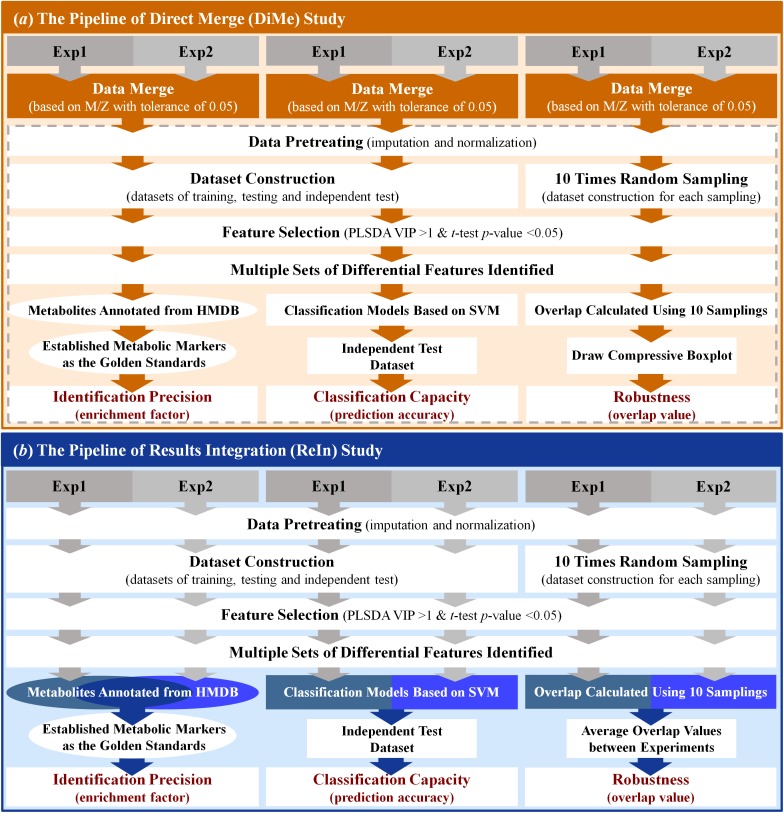
Schematic representations of the workflows of the analytical strategies applied in this study. **(a)** the pipeline of direct merge; **(b)** the pipeline of results integration.

The workflow of the *DiMe* strategy applied in this work was systematically illustrated in Figure 2a. In this study, four pairs of metabolomics benchmark datasets were adopted to assess the performance of *DiMe* strategy, which included the pair of experimental dataset (1) and dataset (2) from MTBLS17 ESI+ (Haug et al., 2013), the pair of experimental dataset (3) and dataset (4) from MTBLS17 ESI- (Haug et al., 2013), the pair of experimental dataset (5) and dataset (6) from MTBLS19 ESI+ (Haug et al., 2013), and the pair of experimental dataset (7), and dataset (8) from MTBLS19 ESI- (Haug et al., 2013). In each experimental dataset, the peak detection, retention time (RT) correction and peak alignment were first applied to the UHPLC/Q-TOF-MS raw data (in CDF format) using the *xcmsSet, group* and *rector* functions in XCMS package (Smith et al., 2006) by setting both *fwhm* and *bw* equal to ten (Li et al., 2016). Then, two datasets in each pair were merged based on their m/z values with tolerance of 0.05 ppm (Zhang et al., 2014). In particular, the common peaks within above tolerance between two datasets was selected, based on which these datasets were merged into a large one.

Prior to the biomarker identification, the datasets were frequently pretreated in current metabolomics study (De Livera et al., 2012; Zhu et al., 2018; Zuo et al., 2018). Herein, the pretreatment of merged dataset was then conducted, which included the missing value imputation using k-Nearest Neighbor (KNN) method and data normalization using MSTUS. The KNN method imputed values based on K features similar to the features with missing values (Shah et al., 2017). Among the available imputation methods, the KNN algorithm was reported as the most robust one for analyzing MS-based metabolomic data (Di Guida et al., 2016). By assuming that the number of increased and decreased metabolic signals is relatively equivalent, the MSTUS adopted the total signal of metabolites that was shared by all samples (Warrack et al., 2009). MSTUS was referred as one of the best choices for overcoming sample variability in urinary metabolomics and was used to identify diagnostic and prognostic biomarkers (Chen et al., 2013; Mathe et al., 2014). Therefore, the KNN algorithm and the MSTUS method were adopted in this study to impute the missing signal of metabolite and transform/normalize the data matrix. After the above preparation, the training, testing and independent test datasets were further constructed based on the random sampling of the merged dataset. These three datasets were prepared for assessing the *identification precision* and *classification capacity* of *DiMe* strategy (described in the last section of “Materials and Methods”). Furthermore, another 10 datasets were generated by the random sampling of half of the merged dataset for 10 times, which were further used for evaluating the *robustness* of *DiMe* strategy (described in the last section of “Materials and Methods”).

After all those steps prepared above, the PLSDA was used to identify the differential metabolic peaks between distinct sample groups within each merged dataset. Particularly, the differential peaks were identified by VIP >1 and *p*-value < 0.05 (Fan et al., 2016), which were subsequently annotated based on human metabolome database (HMDB) (Wishart et al., 2013) by setting m/z tolerance equal to 20 ppm (Peng and Li, 2013). Those resulting metabolites annotated were the metabolic biomarkers finally identified. All in all, the workflow of *DiMe* strategy applied in this study was systematically illustrated in Figure 2a.

### Results Integration (*ReIn*) Strategy Used in This Study Based on the Identified Biomarkers

The workflow of the *ReIn* strategy applied in this work was systematically illustrated in Figure 2b. The same four pairs of metabolomics benchmark datasets as used in *DiMe* strategy were used in this analysis. For the experimental dataset in each pair, peak detection, RT correction and peak alignment were first conducted using the *xcmsSet, group* and *rector* functions in XCMS package (Smith et al., 2006) by setting *fwhm* and *bw* to ten (Li et al., 2016). Second, the pretreatment of each experimental dataset was conducted using KNN for missing value imputation and MSTUS for data normalization. Third, the training, testing and independent test datasets were constructed by random sampling each pretreated experimental dataset. These three datasets were prepared for assessing the *identification precision* and *classification capacity* of the *ReIn* strategy (described in the last section of “Materials and Methods”). Meanwhile, another 10 datasets were generated by the random sampling of half of the pretreated experimental dataset for 10 times, which were applied for the evaluation of *robustness* of the *ReIn* strategy (described in the last section of “Materials and Methods”). Fourth, PLSDA was used to identify the differential metabolic peaks between distinct sample groups within each dataset (VIP>1 and *p*-value < 0.05). The resulting metabolites annotated based on HMDB by setting the m/z tolerance equal to 20 ppm were the metabolic biomarkers finally identified. Finally, the metabolites annotated from two experimental datasets were collectively considered for assessing *identification precision* of the *ReIn* strategy, the classification models constructed based on experimental datasets were integrated for evaluating *ReIn*’s *classification capacity*, and the *robustness* of the *ReIn* strategy was also collectively determined by the average overlap values between two experiments. All in all, the workflow of *ReIn* strategy applied in this study was systematically illustrated in Figure 2b.

### Multiple Criteria Used for the Performance Assessment of the Strategies Applied

Three well-established criteria for the performance assessment of the strategies applied were adopted in this study, which included the *identification precision, classification capacity* and *robustness*. As reported, these three criteria were independent from each other (Li et al., 2017b), which was required to be collectively considered during the performance assessments (Tang et al., 2019). In other words, these three criteria were mutually complemental from different perspectives, and all were important for assessing the performance of the analytical strategy applied in metabolomic studies (Tang et al., 2019). Therefore, all these criteria were adopted in this study for performance assessment.

#### Identification Precision

Recent studies emphasized the importance of the experimentally validated true markers in evaluating the identification precision of analytical strategies (Li et al., 2016; Cai et al., 2017; Li et al., 2017b). These well-established true metabolic markers were then used as a golden standard to assess the identification precision based on the EF (Zhang et al., 2011; Liu et al., 2014). The EF was used to measure the enhanced chances of true marker identification by a given analytical strategy over the random selection of true markers from all metabolites (Zhang et al., 2011; Liu et al., 2014). In this study, a comprehensive literature review on the experimentally validated true markers differentiating HCC patients from those with CIR was first conducted. Then, the EF of each analytical strategy was calculated based on Eq. 1:

(1) $$\mathrm{EF=}\frac{\mathrm{true}\;\mathrm{maker}\;\mathrm{identification}\;\mathrm{rate}\;\mathrm{from}\;\mathrm{all}\;\mathrm{makers}\;\mathrm{identified}}{\mathrm{true}\;\mathrm{maker}\;\mathrm{identification}\;\mathrm{rate}\;\mathrm{by}\;\mathrm{random}\;\mathrm{selection}}$$

EF denoted the level of enhancement in true marker identification rate (Zhang et al., 2011). EF = 1 meant no better than random selection. The larger EF, the greater the likelihood to find true marker.

#### Classification Capacity

Based on three datasets after “dataset construction” (Figure 2), the SVM was first applied to construct the classification model based on both training and testing datasets together with the biomarkers identified by Student’s *t*-test (*p*-value < 0.05). Then, independent test set was used to assess the classification capacity of constructed model, which was evaluated by the ROC analysis together with the measurement of AUC (Kohl et al., 2012). The AUC values were widely considered to be one of the most objective and valid metrics for the performance evaluation of biomarker discovery (Xia et al., 2015). Moreover, the classification capacity was frequently assessed by four popular metrics including the SEN, SPE, accuracy (ACC), MCC. Particularly, SEN was defined by the percentage of true positive samples correctly identified as “positive” (shown in Eq. 2); SPE denoted the proportion of true negative samples that were correctly predicted as “negative” (shown in Eq. 3); ACC indicated the number of true samples (positive plus negative) divided by the number of all studied samples (shown in Eq. 4); MCC reflected the stability of classification capacity, which described the correlation between a predictive value and an actual value (shown in Eq. 5).

(2) $$\mathrm{SEN}=\frac{\mathrm{TP}}{\mathrm{TP}+\mathrm{FN}}$$

(3) $$\mathrm{SPE}=\frac{\mathrm{TN}}{\mathrm{TN}+\mathrm{FP}}$$

(4) $$\mathrm{ACC}=\frac{\mathrm{TP+TN}}{\mathrm{TP+FN+TN}+\mathrm{FP}}$$

(5) $$\mathrm{MCC}=\frac{\mathrm{(TP*TN}-\mathrm{FP}*\mathrm{FN})}{\sqrt{\left(\mathrm{TP}+\mathrm{FN})*(\mathrm{TP}+\mathrm{FP})*(\mathrm{TN}+\mathrm{FP})*(\mathrm{TN+FN}\right)}}$$

where TP, TN, FP, and FN denoted the number of true positive samples, true negative samples, false positive samples and false negative samples, respectively.

#### Robustness

First, ten sub-datasets were generated by the random sampling of half of the pretreated experimental/merged dataset for ten times. Second, the biomarkers were identified using Student’s *t*-test (*p*-value < 0.05) for each dataset, and ten lists of biomarkers were discovered. Third, for any 2 marker lists, the fraction of shared marker appearing on both lists were used to measure the similarity of these two lists. Particularly, *overlap* value was calculated (shown in Eq. 6) based on marker lists a and b. The closer the *overlap* value equal to 1, the more robust the markers discovered in that study (Wang et al., 2014). For each experimental/merged dataset, 45 (C_1_0^2^ ) *overlap* values denoting all possible combinations between any two sub-datasets were thus calculated and analyzed here.

(6) $$\mathrm{overlap}=\frac{2\times \mathrm{interset}\;(a,\;b)}{N_{a}+N_{b}}$$

where a and b indicated two maker lists, and Na and Nb denoted the number of markers in each list.

## Results and Discussion

### Comparative Analysis on the Classification Capacities of the Constructed Models

Classification model was frequently constructed in current metabolomics research to predict samples of different disease states (Date and Kikuchi, 2018; Maudsley et al., 2018) or assess the reliability of identified metabolic markers (Song et al., 2017). The capacities of the constructed classification model were evaluated by various metrics including *ACC, SEN, SPE, MCC, ROC*, and the area under ROC curve (*AUC* value) (Hart et al., 2017; Hou et al., 2018; Yu et al., 2018). As illustrated in Figure 2, four different analytical strategies, including two strategies based on datasets collected from single experiment (SiE1 and SiE2) and two additional strategies of *ReIn* and *DiMe*, were first evaluated by calculating their *ACC, SEN, SPE*, and *MCC*. As shown in Table 1, there was great variation in each assessment metric among four strategies and among four benchmark datasets. Particularly, the *ACCs, SENs, SPEs*, and *MCCs* of MTBLS17-POS were in the ranges of 0.59∼0.80, 0.33∼0.58, 0.59∼0.92, and 0.12∼0.50 among strategies, respectively, and that of *DiMe* was estimated to be within 0.72∼0.80, 0.50∼0.82, 0.77∼1.00, and 0.44∼0.60 among datasets, respectively. The metrics *ACC* and *MCC* were frequently adopted in current metabolomics to evaluate correctness (Alonso et al., 2016) and stability (Wu et al., 2018) of constructed prediction models. As demonstrated in Table 1, the *ACCs* of *DiMe* were in the range of 0.72∼0.80, which were substantially and consistently higher than that of the other 3 strategies (0.56∼0.74). Similar to *ACCs*, the *MCCs* of *DiMe* (0.44∼0.60) were discovered to be robustly higher than that of the other strategies (0.06∼0.32), and the majority (75%) of *DiMe*’s *MCCs* were larger than 0.50.

**Table 1:** Classification capacities of different analytical strategies assessed by accuracy (ACC), sensitivity (SEN), specificity (SPE), Matthews correlation coefficient (MCC) and area under the curve (AUC) based on four pairs of benchmark datasets collected from the *Metabolights* database.

Experiment ID		ACC	SEN	SPE	MCC	AUC
**MTBLS17-NEG**	SiE1	0.74	0.67	0.75	0.32	0.79
	SiE2	0.69	0.33	0.80	0.13	0.60
	*ReIn*	0.73	0.60	0.76	0.29	0.70
	*DiMe*	0.78	0.82	0.77	0.53	0.85

**MTBLS17-POS**	SiE1	0.59	0.58	0.59	0.13	0.57
	SiE2	0.69	0.33	0.80	0.13	0.76
	*ReIn*	0.60	0.53	0.62	0.12	0.66
	*DiMe*	0.80	0.53	0.92	0.50	0.83

**MTBLS19-NEG**	SiE1	0.67	0.50	0.80	0.32	0.80
	SiE2	0.56	0.50	0.60	0.10	0.80
	*ReIn*	0.61	0.50	0.70	0.20	0.80
	*DiMe*	0.78	0.50	1.00	0.60	0.93

**MTBLS19-POS**	SiE1	0.56	0.25	0.80	0.06	0.70
	SiE2	0.67	0.50	0.80	0.32	0.75
	*ReIn*	0.61	0.38	0.80	0.19	0.73
	*DiMe*	0.72	0.50	0.90	0.44	0.88


**FIGURE 3:**
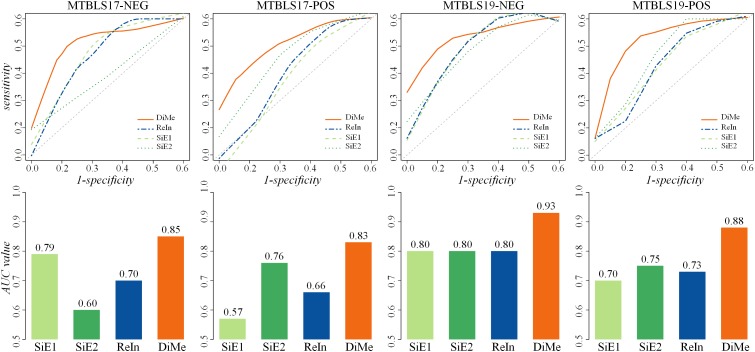
Classification capacities of different analytical strategies assessed by receiver operating characteristic (ROC) and area under the curve (AUC) based on four pairs of benchmark datasets collected from the *Metabolights* database.

Apart from *ACC* and *MCC*, the *ROC* and *AUC* were two other popular metrics widely used to assess classification ability, which were acknowledged to achieve a comprehensive performance evaluation. As illustrated in Figure 3, the *ROC* curves and the *AUC* values of 4 benchmark datasets (MTBLS17-NEG, MTBLS17-POS, MTBLS19-NEG, and MTBLS19-POS) were compared. Two benchmark sets (MTBLS17-NEG and MTBLS17-POS) contained 503 samples (including 358 and 145 patients with liver cirrhosis and HCC, respectively), and the other datasets MTBLS19-NEG and MTBLS19-POS consisted of 180 samples (100 patients with liver cirrhosis and 80 patients with HCC). The gray diagonals represented an invalid model with the corresponding AUC value equaled to 0.5. As shown in Table 1, the *AUC* values of *DiMe* among different datasets (0.82∼0.93) were substantially and consistently higher than that of the other 3 strategies (0.57∼0.80), which were similar to the results assessed by *ROC* curves. In conclusion, this finding indicated that classification correctness (assessed by *ACC, ROC*, and *AUC*) and prediction stability (evaluated by *MCC*) of the direct merge strategy (*DiMe*) were found consistently better across multiple benchmark datasets compared with the SiE1 and SiE2 strategies and the one of results integration (*ReIn*).

### Robustness Assessment of the Markers Identified by Different Analytical Strategies

Apart from prediction capacity evaluated simultaneously by classification correctness and prediction stability, the robustness of identified metabolic markers was widely accepted to be another important metric with underlying theory distinct from that of prediction capacity (Li et al., 2017b; Valikangas et al., 2018). So far, *overlap* value had been recognized as the quantitative measure of the robustness of the identified markers (Wang et al., 2014). The higher *overlap* values represented the more robust metabolic markers identified from a particular dataset by a given strategy. In this study, a sub-dataset was first generated by randomly selecting 50% of both cases and controls in each benchmark dataset, and ten iterations of this selection procedure resulted in ten sub-datasets. For each sub-dataset, a list of differentially expressed metabolic markers were then identified by Student’s *t*-test (*p*-value < 0.05), and the value of *overlap* between any two sub-datasets was calculated using their corresponding lists of markers identified. In total, there were 45 ($C_{10}^{2}$) *overlap* values denoting all possible combinations between any two sub-datasets. Finally, the *overlap* values of four different analytical strategies were compared. As shown in Table 2, the total numbers of markers identified by ten sub-datasets together with the median values of *overlap* were provided. It was obvious that the total numbers of identified markers among ten sub-datasets varied significantly (from 11 to 334). Moreover, although there was great difference among the median *overlap* values (from 0.15 to 0.40), the median *overlap* of *DiMe* was found consistently larger than that of the other three strategies.

**Table 2:** Robustness of different analytical strategies assessed by the number of markers selected by each sampling set and overlap values based on four pairs of benchmark datasets collected from the *Metabolights* database.

Experiment ID		No. of Cases/Controls	No. of MS Peaks Detected	No. of markers selected by the *n*th sampling set										Overlap Median across 10 Samplings
				
				1	2	3	4	5	6	7	8	9	10	
**MTBLS17-N**	SiE1	59/129	941	216	219	74	87	135	276	63	70	42	96	**0.32**
	SiE2	13/50	1,209	37	107	50	135	47	170	60	64	129	64	**0.15**
	*ReIn*	72/179	941/1,209	127	163	62	111	91	223	62	67	86	80	**0.23**
	*DiMe*	72/179	734	145	81	53	115	57	95	57	66	54	125	**0.40**

**MTBLS17-P**	SiE1	60/129	1,586	161	141	43	84	113	43	114	66	114	195	**0.23**
	SiE2	13/50	3,230	128	161	597	179	173	140	291	167	278	233	**0.21**
	*ReIn*	73/179	1,586/3,230	145	151	320	132	143	92	203	117	196	214	**0.19**
	*DiMe*	73/179	1,144	173	68	334	107	82	112	90	106	109	106	**0.36**

**MTBLS19-N**	SiE1	20/25	883	34	51	53	56	39	23	179	73	118	123	**0.27**
	SiE2	20/24	825	27	114	139	216	42	60	22	112	12	32	**0.21**
	*ReIn*	40/50	883/825	31	83	96	136	41	41.5	101	93	65	78	**0.26**
	*DiMe*	40/50	665	66	11	57	187	109	47	27	60	76	37	**0.31**

**MTBLS19-P**	SiE1	20/25	1,526	57	104	63	91	74	164	86	76	37	52	**0.19**
	SiE2	20/24	1,542	229	77	34	187	170	150	80	248	175	57	**0.22**
	*ReIn*	40/50	1,526/1,542	143	91	49	139	122	157	83	162	106	55	**0.23**
	*DiMe*	40/50	872	132	29	110	80	102	148	206	110	163	146	**0.39**


**FIGURE 4:**
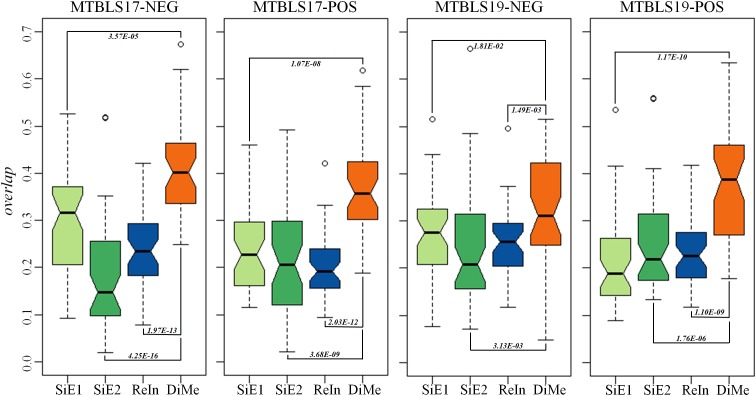
Robustness of different analytical strategies assessed by the overlap values based on four pairs of benchmark datasets collected from the *Metabolights* database.

Compared with the median value of *overlap*, the statistical difference of 45 *overlap* values between different analytical strategies was more meaningful to reveal the level of robustness for each strategy. Thus, comprehensive statistical comparison of robustness among different strategies was conducted and illustrated in Figure 4. The *overlap* values of SiE1, SiE2, *ReIn*, and *DiMe* were colored in light green, dark green, blue, and orange, respectively. Apart from the enhanced median values of *overlap* by *DiMe*, all overlap values of *DiMe* were found statistically higher (*p*-value < 0.05) compared with that of the other strategies. In particular, as illustrated in Figure 4, the statistical differences between *DiMe* and other strategies (*p*-value) were always lower than 0.05 within the range from 4.25E-16 to 1.81E-02. Moreover, the majority of the *overlap* values of *DiMe* were larger than 0.3, while that of the other strategies were lower than 0.3. These findings indicated that the *DiMe* strategy performed better than others in the robustness of the identified markers. Additionally, Table 3 demonstrated the information of markers simultaneously discovered by N (*N* ≥ 6, ≥ 7, ≥ 8, ≥ 9, = 10) sub-datasets, which included the number and percentage of markers co-identified by these N datasets. It was very clearly to see that the robustness of metabolic markers identified by *DiMe* was much better than other three strategies in terms of both the number and the percentage of co-identified markers. Particularly, the percentages of markers identified by over five sub-datasets using *DiMe* were within 3.25%∼5.07%, while that using SiE1 and SiE2 were 0.87%∼2.74% and 0.93%∼2.06%, respectively. Moreover, the percentages of markers identified by all sub-datasets using *DiMe* were within 0.00%∼0.41%, while that using SiE1 and SiE2 were 0.00%∼0.25% and 0.00%∼0.21%, respectively.

**Table 3:** Robustness of different analytical strategies assessed by the percent and number of markers discovered simultaneously by multiple sampling datasets based on four pairs of benchmark datasets collected from the *Metabolights* database.

	MTBLS17-NEG			MTBLS17-POS			MTBLS19-NEG			MTBLS19-POS		
				
	SiE1	SiE2	*DiMe*	SiE1	SiE2	*DiMe*	SiE1	SiE2	*DiMe*	SiE1	SiE2	*DiMe*
**No. of markers identified**	**1,278**	**863**	**848**	**1,074**	**2,347**	**1,287**	**749**	**776**	**677**	**804**	**1,407**	**1,226**

**Percent (No.) of makers discovered simultaneously by N datasets (*N* = )**												
10	0.00% (0)	0.00% (0)	0.12% (1)	0.09% (1)	0.00% (0)	0.31% (4)	0.00% (0)	0.00% (0)	0.00% (0)	0.25% (2)	0.21% (3)	0.41% (5)
≥9	0.31% (4)	0.00% (0)	1.30% (11)	0.19% (2)	0.09% (2)	1.01% (13)	0.80% (6)	0.00% (0)	1.18% (8)	0.37% (3)	0.36% (5)	1.14% (14)
≥8	0.70% (9)	0.35% (3)	2.48% (21)	0.56% (6)	0.34% (8)	1.55% (20)	1.20% (9)	0.13% (1)	1.77% (12)	0.37% (3)	0.50% (7)	1.55% (19)
≥7	1.17% (15)	0.35% (3)	4.01% (34)	0.93% (10)	0.64% (15)	2.87% (37)	1.34% (10)	0.52% (4)	2.07% (14)	0.62% (5)	1.07% (15)	2.45% (30)
≥6	2.74% (35)	0.93% (8)	5.07% (43)	2.05% (22)	1.15% (27)	3.81% (49)	2.00% (15)	1.29% (10)	3.25% (22)	0.87% (7)	2.06% (29)	4.24% (52)


### Evaluation on the False Discovery Rates by Experimentally Validated True Markers

Recent studies emphasized the importance of spike-in metabolites and experimentally validated true markers in evaluating the false discovery rates of analytical strategy (Li et al., 2016; Cai et al., 2017; Li et al., 2017b). These well-established true metabolic markers were frequently used as the golden standard to assess the false discovery rates based on their identification EF (Zhang et al., 2011; Liu et al., 2014). Hence, a comprehensive literature review on the experimentally validated true markers differentiating HCC patients from those with CIR was first conducted in this study. As a result, thirteen discriminative markers between HCC and CIR patients were identified (Table 4). As shown, some metabolic markers (like *glycochenodeoxycholic acid*) were identified from serum samples combining TOF MS/MS with UPLC-SRM-MS/MS based on the internal standard isotope dilution (Tan et al., 2012; Xiao et al., 2012; Kimhofer et al., 2015), and some other markers (like *16:0 lysophosphatidic acid* and *phenylalanine*) were detected by the targeted analysis based on UPLC-ESI-TQMS (Patterson et al., 2011) and LC-MRM-MS/MS (Baniasadi et al., 2013). *Carnitine* and *creatinine* were first discovered by analyzing urinary 1H MRS data (Shariff et al., 2010), but *carnitine* was also identified as true marker in serum samples (Xiao et al., 2012). Since the four benchmark datasets analyzed in this study were serum-based data, these experimentally validated true metabolic markers (twelve biomarkers in total, except *creatinine*, Table 4) were therefore used here to evaluate the false discovery rates of each analytical strategy.

**Table 4:** A variety of metabolite biomarkers differentiating the patients of hepatocellular carcinoma (HCC) from those of cirrhosis (CIR) identified during the past ten years.

No.	True metabolite markers differentiating HCC and CIR	HMDB ID	Bio-fluid used for marker identification	Experimental strategy applied for marker identification	Reference
1	16:0 lysophosphatidic acid	10382	Serum	Profiled and then identified by UPLC-ESI-TQMS based on the internal metabolite standard	Patterson et al., 2011
2	18:0 lysophosphatidic acid	10384	Serum	Combining the TOF MS/MS with UPLC-SRM-MS/MS using internal standard-based isotope dilution	Kimhofer et al., 2015
3	Acetyl carnitine	00201	Serum/Urine	Verified by acquiring MS/MS spectra and further confirmed based on the structure of commercial standard	Lu et al., 2016
4	Carnitine	00562	Serum/Urine	Discovered by serum-based isotope dilution using LC-MS/MS and analyzing the urine-based 1H MRS data	Xiao et al., 2012
5	Creatinine	00062	Urine	Identified experimentally by statistically analyzing the urine-based 1H MRS data	Shariff et al., 2010
6	Glycochenodeoxycholic acid	00637	Serum	Verified by acquiring MS/MS spectra and then quantified using internal standard-based isotope dilution by UPLC-MS/MS	Ressom et al., 2012
7	Glycocholic acid	00138	Serum	Verified by acquiring MS/MS spectra and then quantified using internal standard-based isotope dilution by UPLC-MS/MS	Ressom et al., 2012
8	Glycodeoxycholic acid	00631	Serum	Discovered by the serum-based isotope dilution integrating the internal standard with UPLC-SRM-MS/MS	Xiao et al., 2012
9	Oleamide	02117	Serum	Experimentally validated and identified by UPLC-MS profiling of serum-based data	Jee et al., 2018
10	Phenylalanine	00159	Serum	Detected from the serum samples based on the targeted analysis using LC-MRM-MS/MS	Baniasadi et al., 2013
11	Phenylalanyl-tryptophan	29006	Serum	Identified by the targeted profiling using serum-based UPLC-MS and determined by isotope-labeled quantification	Luo et al., 2017
12	Taurochenodeoxycholic acid	00951	Serum	Discovered by the serum-based isotope dilution integrating the internal standard with UPLC-SRM-MS/MS	Xiao et al., 2012
13	Taurocholic acid	00036	Serum	Verified by acquiring MS/MS spectra and then quantified using internal standard-based isotope dilution by UPLC-MS/MS	Ressom et al., 2012


*ESI: electrospray ionization; MRM: multiple reaction monitoring; MRS: magnetic resonance spectroscopy; SRM: selected reaction monitoring; TOF: time-of-flight; TQMS: triple quadrupole mass spectrometry; UPLC: ultraperformance liquid chromatography.*

Table 5 provided the number of the true makers covered by both detected and identified metabolites. For each experimental dataset (MTBLS17-NEG, MTBLS17-POS, MTBLS19-NEG, and MTBLS19-POS), there were variations in their number of true markers covered by the detected metabolites. In particular, the detected metabolites in MTBLS17-POS contained the highest number of true markers (11 for all strategies) and that in MTBLS17-NEG covered the most variated numbers of true markers among four strategies (from 5 to 9). Furthermore, the number of true markers identified by strategies SiE1 and SiE2 was found to be basically no less than that of *ReIn* and *DiMe*, which represented the relatively equal abilities in true marker identification among different strategies. However, as shown in Table 5, the EF of both SiE1 and SiE2 was consistently lower than that of *ReIn* and *DiMe*, which indicated that, compared with *ReIn* and *DiMe*, the total numbers of true markers discovered by SiE1 and SiE2 were more at the cost of discovering numerous false metabolites. Moreover, among those integration/merging-based strategies (*ReIn* and *DiMe*), the EF values of *ReIn* in three experimental datasets (MTBLS17-POS, MTBLS19-NEG, and MTBLS19-POS) were found to be obviously higher than those of *DiMe* strategy, which reflected the superior ability of *ReIn* strategy in controlling false discovery rate. However, in one extreme case (MTBLS17-NEG), the EF of *ReIn* was lower than that of *DiMe*. Careful investigation of Table 5 revealed that only one true marker was identified by *ReIn*, which led to a huge decline in its EF values. Therefore, although *ReIn* demonstrated superior ability to control false discovery rate, its application could be limited by its relatively small number of true markers identified.

**Table 5:** False discovery rate of different analytical strategies assessed by the number of true markers identified and the enrichment factor (EF) based on four pairs of benchmark datasets collected from the *Metabolights* database.

Experiment ID		No. of cases / controls	No. of MS peaks detected	No. of metabolites annotated based on detected peaks	No. of true markers covered by detected metabolites	No. of differential peaks identified	No. of metabolites annotated based on identified peaks	No. of true markers covered by identified metabolites	Enrichment factor
**MTBLS17-NEG**	SiE1	59/129	941	42,269	9	172	9709	5	**2.42**
	SiE2	13/50	1,209	43,614	8	174	3296	2	**3.31**
	*ReIn*	72/179	941/1,209	32,592	5	-	930	1	**7.01**
	*DiMe*	72/179	734	34,840	7	141	2523	4	**7.89**

**MTBLS17-POS**	SiE1	60/129	1,586	19,724	11	205	6760	7	**1.86**
	SiE2	13/50	3,230	24,157	11	215	5815	5	**1.89**
	*ReIn*	73/179	1,586/3,230	19,724	11	-	1862	5	**4.81**
	*DiMe*	73/179	1,144	19,272	11	182	5503	7	**2.23**

**MTBLS19-NEG**	SiE1	20/25	883	28,088	7	122	5163	3	**2.33**
	SiE2	20/25	825	25,950	7	135	7049	4	**2.10**
	*ReIn*	40/50	883/825	22,992	7	-	1307	2	**5.03**
	*DiMe*	40/50	665	23,040	7	107	1931	2	**3.41**

**MTBLS19-POS**	SiE1	20/25	1,526	17,166	9	202	4205	5	**2.26**
	SiE2	20/25	1,542	17,966	8	202	6028	4	**1.49**
	*ReIn*	40/50	1,526/1,542	15,215	8	-	1789	4	**4.25**
	*DiMe*	40/50	872	14,935	8	82	2469	3	**2.27**


## Conclusion

Based on the systematic review of *MetaboLights*, a comprehensive evaluation of different analytical strategies was conducted by assessing the classification capacity, robustness and false discovery rate. As a result, the integration/merging-based strategies (*ReIn* & *DiMe*) performed better than strategies based on single experiment (SiE1 & SiE2). Moreover, *DiMe* strategy was found to outperform *ReIn* in *classification capacity* and *robustness*, while *ReIn* demonstrated superior capacity in controlling false discovery rate. In summary, these findings may facilitate current metabolomics study in *classification capacity, identification precision*, and *robustness*.

## Author Contributions

FZ conceived the idea and supervised the work. XC, QY, and BL performed the research. XC, QY, BL, JT, XZ, SL, FL, JH, YL, YQ, and WX prepared the program and analyzed the data. FZ and JH wrote the manuscript. All authors have read and approved this manuscript.

## Conflict of Interest Statement

The authors declare that the research was conducted in the absence of any commercial or financial relationships that could be construed as a potential conflict of interest.

## Abbreviations

AUC: area under the curve
CIR: cirrhosis
*DiMe*: direct data merging
EF: enrichment factor
ESI: electrospray ionization
HCC: hepatocellular carcinoma
KNN: k-nearest neighbors
LC-MS: liquid chromatography-mass spectrometry
MCC: matthews correlation coefficient
MRM-MS: multiple reaction monitoring mass spectrometry
MSTUS: mass spectrum total useful signal
PLSDA: partial least squares discrimination analysis
*ReIn*: integration of the results
ROC: receiver operating characteristic
SEN: sensitivity
SPE: specificity
SVM: Support Vector Machine
UPLC-QTOF MS: ultra-high performance liquid chromatography-quadrupole time-of-flight mass spectrometry
VIP: Variable Importance in the Projection

## References

[B1] Alonso A. Julia A. Vinaixa M. Domenech E. Fernandez-Nebro A. Canete J. D. (2016). Urine metabolome profiling of immune-mediated inflammatory diseases. *BMC Med.* 14:133. 10.1186/s12916-016-0681-8 27609333 PMC5016926

[B2] Baniasadi H. Gowda G. A. Gu H. Zeng A. Zhuang S. Skill N. (2013). Targeted metabolic profiling of hepatocellular carcinoma and hepatitis C using LC-MS/MS. *Electrophoresis* 34 2910–2917. 10.1002/elps.201300029 23856972 PMC3826436

[B3] Billoir E. Navratil V. Blaise B. J. (2015). Sample size calculation in metabolic phenotyping studies. *Brief. Bioinform.* 16 813–819. 10.1093/bib/bbu052 25600654

[B4] Brunius C. Shi L. Landberg R. (2016). Large-scale untargeted LC-MS metabolomics data correction using between-batch feature alignment and cluster-based within-batch signal intensity drift correction. *Metabolomics* 12:173. 10.1007/s11306-016-1124-4 27746707 PMC5031781

[B5] Button K. S. Ioannidis J. P. Mokrysz C. Nosek B. A. Flint J. Robinson E. S. (2013). Power failure: why small sample size undermines the reliability of neuroscience. *Nat. Rev. Neurosci.* 14 365–376. 10.1038/nrn3475 23571845

[B6] Cai J. Zhang J. Tian Y. Zhang L. Hatzakis E. Krausz K. W. (2017). Orthogonal comparison of GC-MS and (1)H NMR spectroscopy for short chain fatty acid quantitation. *Anal. Chem.* 89 7900–7906. 10.1021/acs.analchem.7b00848 28650151 PMC6334302

[B7] Chen C. Wang F. Xiao W. Xia Z. Hu G. Wan J. (2017). Effect on platelet aggregation activity: extracts from 31 traditional chinese medicines with the property of activating blood and resolving stasis. *J. Tradit. Chin. Med.* 37 64–75. 10.1016/S0254-6272(17)30028-6 29957905

[B8] Chen Y. Shen G. Zhang R. He J. Zhang Y. Xu J. (2013). Combination of injection volume calibration by creatinine and MS signals’ normalization to overcome urine variability in LC-MS-based metabolomics studies. *Anal. Chem.* 85 7659–7665. 10.1021/ac401400b 23855648

[B9] Date Y. Kikuchi J. (2018). Application of a deep neural network to metabolomics studies and its performance in determining important variables. *Anal. Chem.* 90 1805–1810. 10.1021/acs.analchem.7b03795 29278490

[B10] De Livera A. M. Dias D. A. De Souza D. Rupasinghe T. Pyke J. Tull D. (2012). Normalizing and integrating metabolomics data. *Anal. Chem.* 84 10768–10776. 10.1021/ac302748b 23150939

[B11] Di Guida R. Engel J. Allwood J. W. Weber R. J. Jones M. R. Sommer U. (2016). Non-targeted UHPLC-MS metabolomic data processing methods: a comparative investigation of normalisation, missing value imputation, transformation and scaling. *Metabolomics* 12:93. 10.1007/s11306-016-1030-9 27123000 PMC4831991

[B12] Fan Y. Li Y. Chen Y. Zhao Y. J. Liu L. W. Li J. (2016). Comprehensive metabolomic characterization of coronary artery diseases. *J. Am. Coll. Cardiol.* 68 1281–1293. 10.1016/j.jacc.2016.06.044 27634119

[B13] Fu J. Tang J. Wang Y. Cui X. Yang Q. Hong J. (2018). Discovery of the consistently well-performed analysis chain for SWATH-MS based pharmacoproteomic quantification. *Front. Pharmacol.* 9:681. 10.3389/fphar.2018.00681 29997509 PMC6028727

[B14] Goveia J. Pircher A. Conradi L. C. Kalucka J. Lagani V. Dewerchin M. (2016). Meta-analysis of clinical metabolic profiling studies in cancer: challenges and opportunities. *EMBO Mol. Med.* 8 1134–1142. 10.15252/emmm.201606798 27601137 PMC5048364

[B15] Guasch-Ferre M. Hruby A. Toledo E. Clish C. B. Martinez-Gonzalez M. A. Salas-Salvado J. (2016). Metabolomics in prediabetes and diabetes: a systematic review and meta-analysis. *Diabetes Care* 39 833–846. 10.2337/dc15-2251 27208380 PMC4839172

[B16] Han Z. J. Xue W. W. Tao L. Zhu F. (2018). Identification of novel immune-relevant drug target genes for alzheimer’s disease by combining ontology inference with network analysis. *CNS Neurosci. Ther.* 24 1253–1263. 10.1111/cns.13051 30106219 PMC6489817

[B17] Hart C. D. Vignoli A. Tenori L. Uy G. L. Van To T. Adebamowo C. (2017). Serum metabolomic profiles identify ER-positive early breast cancer patients at increased risk of disease recurrence in a multicenter population. *Clin. Cancer Res.* 23 1422–1431. 10.1158/1078-0432.CCR-16-1153 28082280 PMC5695865

[B18] Haug K. Salek R. M. Conesa P. Hastings J. de Matos P. Rijnbeek M. (2013). MetaboLights–an open-access general-purpose repository for metabolomics studies and associated meta-data. *Nucleic Acids Res.* 41 D781–D786. 10.1093/nar/gks1004 23109552 PMC3531110

[B19] He J. Wang K. Zheng N. Qiu Y. Xie G. Su M. (2015). Metformin suppressed the proliferation of LoVo cells and induced a time-dependent metabolic and transcriptional alteration. *Sci. Rep.* 5:17423. 10.1038/srep17423 26616174 PMC4663508

[B20] Hou W. Meng X. Zhao A. Zhao W. Pan J. Tang J. (2018). Development of multimarker diagnostic models from metabolomics analysis for gestational diabetes mellitus (GDM). *Mol. Cell. Proteomics* 17 431–441. 10.1074/mcp.RA117.000121 29282297 PMC5836369

[B21] Hu X. Shen J. Pu X. Zheng N. Deng Z. Zhang Z. (2017). Urinary time- or dose-dependent metabolic biomarkers of aristolochic acid-induced nephrotoxicity in rats. *Toxicol. Sci.* 156 123–132. 10.1093/toxsci/kfw244 28115647

[B22] Jee S. H. Kim M. Kim M. Yoo H. J. Kim H. Jung K. J. (2018). Metabolomics profiles of hepatocellular carcinoma in a korean prospective cohort: the korean cancer prevention study-II. *Cancer Prev. Res.* 11 303–312. 10.1158/1940-6207.CAPR-17-0249 29500188

[B23] Kale N. S. Haug K. Conesa P. Jayseelan K. Moreno P. Rocca-Serra P. (2016). MetaboLights: an open-access database repository for metabolomics data. *Curr. Protoc. Bioinformatics* 53 14.13.1–18. 10.1002/0471250953.bi1413s53 27010336

[B24] Kimhofer T. Fye H. Taylor-Robinson S. Thursz M. Holmes E. (2015). Proteomic and metabonomic biomarkers for hepatocellular carcinoma: a comprehensive review. *Br. J. Cancer* 112 1141–1156. 10.1038/bjc.2015.38 25826224 PMC4385954

[B25] Kohl S. M. Klein M. S. Hochrein J. Oefner P. J. Spang R. Gronwald W. (2012). State-of-the art data normalization methods improve NMR-based metabolomic analysis. *Metabolomics* 8 146–160. 10.1007/s11306-011-0350-z 22593726 PMC3337420

[B26] Larsson O. Wennmalm K. Sandberg R. (2006). Comparative microarray analysis. *OMICS* 10 381–397. 10.1089/omi.2006.10.381 17069515

[B27] Lazar C. Meganck S. Taminau J. Steenhoff D. Coletta A. Molter C. (2013). Batch effect removal methods for microarray gene expression data integration: a survey. *Brief. Bioinform.* 14 469–490. 10.1093/bib/bbs037 22851511

[B28] Lee S. Smith C. A. (2012). Criteria for quantitative and qualitative data integration: mixed-methods research methodology. *Comput. Inform. Nurs.* 30 251–256. 10.1097/NXN.0b013e31824b1f96 22411415

[B29] Li B. He X. Jia W. Li H. (2017a). Novel applications of metabolomics in personalized medicine: a mini-review. *Molecules* 22:1173. 10.3390/molecules22071173 28703775 PMC6152045

[B30] Li B. Tang J. Yang Q. Li S. Cui X. Li Y. (2017b). Noreva: normalization and evaluation of MS-based metabolomics data. *Nucleic Acids Res.* 45 W162–W170. 10.1093/nar/gkx449 28525573 PMC5570188

[B31] Li C. H. Chen C. Zhang Q. Tan C. N. Hu Y. J. Li P. (2017c). Differential proteomic analysis of platelets suggested target-related proteins in rabbit platelets treated with *Rhizoma Corydalis*. *Pharm. Biol.* 55 76–87. 10.1080/13880209.2016.1229340 27653279 PMC7011957

[B32] Li B. Tang J. Yang Q. Cui X. Li S. Chen S. (2016). Performance evaluation and online realization of data-driven normalization methods used in LC/MS based untargeted metabolomics analysis. *Sci. Rep.* 6:38881. 10.1038/srep38881 27958387 PMC5153651

[B33] Li W. Dai C. Kang S. Zhou X. J. (2014). Integrative analysis of many RNA-seq datasets to study alternative splicing. *Methods* 67 313–324. 10.1016/j.ymeth.2014.02.024 24583115 PMC4120771

[B34] Li X. Li X. Li Y. Yu C. Xue W. Hu J. (2018). What makes species productive of anti-cancer drugs? Clues from drugs’ species origin, druglikeness, target and pathway. *Anticancer Agents Med. Chem.* 10.2174/1871520618666181029132017 [Epub ahead of print]. 30370862

[B35] Li X. X. Yin J. Tang J. Li Y. Yang Q. Xiao Z. (2018). Determining the balance between drug efficacy and safety by the network and biological system profile of its therapeutic target. *Front. Pharmacol.* 9:1245. 10.3389/fphar.2018.01245 30429792 PMC6220079

[B36] Li Y. H. Yu C. Y. Li X. X. Zhang P. Tang J. Yang Q. (2018). Therapeutic target database update 2018: enriched resource for facilitating bench-to-clinic research of targeted therapeutics. *Nucleic Acids Res.* 46 D1121–D1127. 10.1093/nar/gkx1076 29140520 PMC5753365

[B37] Li Y. H. Li X. X. Hong J. J. Wang Y. X. Fu J. B. Yang H. (2019). Clinical trials, progression-speed differentiating features and swiftness rule of the innovative targets of first-in-class drugs. *Brief. Bioinform.* 10.1093/bib/bby130 [Epub ahead of print]. 30689717 PMC7299286

[B38] Liu T. Diao J. Di S. Zhou Z. (2014). Stereoselective bioaccumulation and metabolite formation of triadimefon in Tubifex tubifex. *Environ. Sci. Technol.* 48 6687–6693. 10.1021/es5000287 24846121

[B39] Lu Y. Li N. Gao L. Xu Y. J. Huang C. Yu K. (2016). Acetylcarnitine is a candidate diagnostic and prognostic biomarker of hepatocellular carcinoma. *Cancer Res.* 76 2912–2920. 10.1158/0008-5472.CAN-15-3199 26976432

[B40] Luo P. Yin P. Hua R. Tan Y. Li Z. Qiu G. et al. (2017). A large-scale, multicenter serum metabolite biomarker identification study for the early detection of hepatocellular carcinoma. Hepatology 10.1002/hep.29561 28960374 PMC6680350

[B41] Mathe E. A. Patterson A. D. Haznadar M. Manna S. K. Krausz K. W. Bowman E. D. (2014). Noninvasive urinary metabolomic profiling identifies diagnostic and prognostic markers in lung cancer. *Cancer Res.* 74 3259–3270. 10.1158/0008-5472.CAN-14-0109 24736543 PMC4100625

[B42] Maudsley S. Devanarayan V. Martin B. Geerts H. Brain Health Modeling I. (2018). Intelligent and effective informatic deconvolution of “Big Data” and its future impact on the quantitative nature of neurodegenerative disease therapy. *Alzheimers Dement.* 14 961–975. 10.1016/j.jalz.2018.01.014 29551332

[B43] Paglia G. Astarita G. (2017). Metabolomics and lipidomics using traveling-wave ion mobility mass spectrometry. *Nat. Protoc.* 12 797–813. 10.1038/nprot.2017.013 28301461

[B44] Patterson A. D. Maurhofer O. Beyoglu D. Lanz C. Krausz K. W. Pabst T. (2011). Aberrant lipid metabolism in hepatocellular carcinoma revealed by plasma metabolomics and lipid profiling. *Cancer Res.* 71 6590–6600. 10.1158/0008-5472.CAN-11-0885 21900402 PMC3206149

[B45] Peng J. Li L. (2013). Liquid-liquid extraction combined with differential isotope dimethylaminophenacyl labeling for improved metabolomic profiling of organic acids. *Anal. Chim. Acta.* 803 97–105. 10.1016/j.aca.2013.07.045 24216202

[B46] Ressom H. W. Xiao J. F. Tuli L. Varghese R. S. Zhou B. Tsai T. H. (2012). Utilization of metabolomics to identify serum biomarkers for hepatocellular carcinoma in patients with liver cirrhosis. *Anal. Chim. Acta* 19 90–100. 10.1016/j.aca.2012.07.013 22882828 PMC3419576

[B47] Shah J. S. Rai S. N. DeFilippis A. P. Hill B. G. Bhatnagar A. Brock G. N. (2017). Distribution based nearest neighbor imputation for truncated high dimensional data with applications to pre-clinical and clinical metabolomics studies. *BMC Bioinformatics* 18:114. 10.1186/s12859-017-1547-6 28219348 PMC5319174

[B48] Shariff M. I. Ladep N. G. Cox I. J. Williams H. R. Okeke E. Malu A. (2010). Characterization of urinary biomarkers of hepatocellular carcinoma using magnetic resonance spectroscopy in a Nigerian population. *J. Proteome Res.* 9 1096–1103. 10.1021/pr901058t 19968328

[B49] Smith C. A. Want E. J. O’Maille G. Abagyan R. Siuzdak G. (2006). XCMS: processing mass spectrometry data for metabolite profiling using nonlinear peak alignment, matching, and identification. *Anal. Chem.* 78 779–787. 10.1021/ac051437y 16448051

[B50] Song L. Zhuang P. Lin M. Kang M. Liu H. Zhang Y. (2017). Urine metabonomics reveals early biomarkers in diabetic cognitive dysfunction. *J. Proteome Res.* 16 3180–3189. 10.1021/acs.jproteome.7b00168 28722418

[B51] Soto-Iglesias D. Butakoff C. Andreu D. Fernandez-Armenta J. Berruezo A. Camara O. (2016). Integration of electro-anatomical and imaging data of the left ventricle: an evaluation framework. *Med. Image Anal.* 32 131–144. 10.1016/j.media.2016.03.010 27086166

[B52] Subramanian S. (2016). The effects of sample size on population genomic analyses–implications for the tests of neutrality. *BMC Genomics* 17:123. 10.1186/s12864-016-2441-8 26897757 PMC4761153

[B53] Switnicki M. P. Juul M. Madsen T. Sorensen K. D. Pedersen J. S. (2016). PINCAGE: probabilistic integration of cancer genomics data for perturbed gene identification and sample classification. *Bioinformatics* 32 1353–1365. 10.1093/bioinformatics/btv758 26740525

[B54] Tan Y. Yin P. Tang L. Xing W. Huang Q. Cao D. (2012). Metabolomics study of stepwise hepatocarcinogenesis from the model rats to patients: potential biomarkers effective for small hepatocellular carcinoma diagnosis. *Mol. Cell. Proteomics* 11:M111010694. 10.1074/mcp.M111.010694 22084000 PMC3277755

[B55] Tang J. Fu J. Wang Y. Li B. Li Y. Yang Q. (2019). Anpela: analysis and performance assessment of the label-free quantification workflow for metaproteomic studies. *Brief. Bioinform.* 10 bby127. 10.1093/bib/bby127 30649171 PMC7299298

[B56] Tang J. Zhang Y. Fu J. Wang Y. Li Y. Yang Q. (2018). Computational advances in the label-free quantification of cancer proteomics data. *Curr. Pharm. Des.* 24 3842–3858. 10.2174/1381612824666181102125638 30387388

[B57] Valikangas T. Suomi T. Elo L. L. (2018). A systematic evaluation of normalization methods in quantitative label-free proteomics. *Brief. Bioinform.* 19 1–11. 10.1093/bib/bbw095 27694351 PMC5862339

[B58] Wang C. Gong B. Bushel P. R. Thierry-Mieg J. Thierry-Mieg D. Xu J. (2014). The concordance between RNA-seq and microarray data depends on chemical treatment and transcript abundance. *Nat. Biotechnol.* 32 926–932. 10.1038/nbt.3001 25150839 PMC4243706

[B59] Wang P. Fu T. Zhang X. Yang F. Zheng G. Xue W. (2017a). Differentiating physicochemical properties between NDRIs and sNRIs clinically important for the treatment of ADHD. *Biochim. Biophys. Acta Gen. Subj.* 1861 2766–2777. 10.1016/j.bbagen.2017.07.022 28757337

[B60] Wang P. Zhang X. Fu T. Li S. Li B. Xue W. (2017b). Differentiating physicochemical properties between addictive and nonaddictive ADHD drugs revealed by molecular dynamics simulation studies. *ACS Chem. Neurosci.* 8 1416–1428. 10.1021/acschemneuro.7b00173 28557437

[B61] Warrack B. M. Hnatyshyn S. Ott K. H. Reily M. D. Sanders M. Zhang H. (2009). Normalization strategies for metabonomic analysis of urine samples. *J. Chromatogr. B Analyt. Technol. Biomed. Life Sci.* 877 547–552. 10.1016/j.jchromb.2009.01.007 19185549

[B62] Wishart D. S. Jewison T. Guo A. C. Wilson M. Knox C. Liu Y. (2013). HMDB 3.0–The human metabolome database in 2013. *Nucleic Acids Res.* 41 D801–D807. 10.1093/nar/gks1065 23161693 PMC3531200

[B63] Wu F. Chi L. Ru H. Parvez F. Slavkovich V. Eunus M. (2018). Arsenic exposure from drinking water and urinary metabolomics: associations and long-term reproducibility in bangladesh adults. *Environ. Health Perspect.* 126:017005. 10.1289/EHP1992 29329102 PMC6014710

[B64] Xia J. Sinelnikov I. V. Han B. Wishart D. S. (2015). MetaboAnalyst 3.0–making metabolomics more meaningful. *Nucleic Acids Res.* 43 W251–W257. 10.1093/nar/gkv380 25897128 PMC4489235

[B65] Xiao J. F. Varghese R. S. Zhou B. Nezami Ranjbar M. R. Zhao Y. Tsai T. H. (2012). LC-MS based serum metabolomics for identification of hepatocellular carcinoma biomarkers in egyptian cohort. *J. Proteome Res.* 11 5914–5923. 10.1021/pr300673x 23078175 PMC3719870

[B66] Xue W. Wang P. Tu G. Yang F. Zheng G. Li X. (2018a). Computational identification of the binding mechanism of a triple reuptake inhibitor amitifadine for the treatment of major depressive disorder. *Phys. Chem. Chem. Phys.* 20 6606–6616. 10.1039/c7cp07869b 29451287

[B67] Xue W. Yang F. Wang P. Zheng G. Chen Y. Yao X. (2018b). What contributes to serotonin-norepinephrine reuptake inhibitors’ dual-targeting mechanism? The key role of transmembrane domain 6 in human serotonin and norepinephrine transporters revealed by molecular dynamics simulation. *ACS Chem. Neurosci.* 9 1128–1140. 10.1021/acschemneuro.7b00490 29300091

[B68] Yang H. Qin C. Li Y. H. Tao L. Zhou J. Yu C. Y. (2016). Therapeutic target database update 2016: enriched resource for bench to clinical drug target and targeted pathway information. *Nucleic Acids Res.* 44 D1069–D1074. 10.1093/nar/gkv1230 26578601 PMC4702870

[B69] Yang Q. Wang Y. Zhang S. Tang J. Li F. Yin J. (2019). Biomarker discovery for immunotherapy of pituitary adenomas: enhanced robustness and prediction ability by modern computational tools. *Int. J. Mol. Sci.* 20:151. 10.3390/ijms20010151 30609812 PMC6337483

[B70] Yu C. Y. Li X. X. Yang H. Li Y. H. Xue W. W. Chen Y. Z. (2018). Assessing the performances of protein function prediction algorithms from the perspectives of identification accuracy and false discovery rate. *Int. J. Mol. Sci.* 19:183. 10.3390/ijms19010183 29316706 PMC5796132

[B71] Zhang A. Sun H. Wang X. (2018). Mass spectrometry-driven drug discovery for development of herbal medicine. *Mass Spectrom. Rev.* 37 307–320. 10.1002/mas.21529 28009933

[B72] Zhang H. H. Ahn J. Lin X. Park C. (2006). Gene selection using support vector machines with non-convex penalty. *Bioinformatics* 22 88–95. 10.1093/bioinformatics/bti736 16249260

[B73] Zhang W. Chang J. Lei Z. Huhman D. Sumner L. W. Zhao P. X. (2014). MET-COFEA: a liquid chromatography/mass spectrometry data processing platform for metabolite compound feature extraction and annotation. *Anal. Chem.* 86 6245–6253. 10.1021/ac501162k 24856452

[B74] Zhang W. Zhao C. Wang S. Fang C. Xu Y. Lu H. (2011). Coating cells with cationic silica-magnetite nanocomposites for rapid purification of integral plasma membrane proteins. *Proteomics* 11 3482–3490. 10.1002/pmic.201000211 21751343

[B75] Zhao Y. Hao Z. Zhao C. Zhao J. Zhang J. Li Y. (2016). A novel strategy for large-scale metabolomics study by calibrating gross and systematic errors in gas chromatography-mass spectrometry. *Anal. Chem.* 88 2234–2242. 10.1021/acs.analchem.5b03912 26757347

[B76] Zheng G. Yang F. Fu T. Tu G. Chen Y. Yao X. (2018). Computational characterization of the selective inhibition of human norepinephrine and serotonin transporters by an escitalopram scaffold. *Phys. Chem. Chem. Phys.* 20 29513–29527. 10.1039/c8cp06232c 30457616

[B77] Zhu F. Han L. Zheng C. Xie B. Tammi M. T. Yang S. (2009). What are next generation innovative therapeutic targets? Clues from genetic, structural, physicochemical, and systems profiles of successful targets. *J. Pharmacol. Exp. Ther.* 330 304–315. 10.1124/jpet.108.149955 19357322

[B78] Zhu F. Li X. X. Yang S. Y. Chen Y. Z. (2018). Clinical success of drug targets prospectively predicted by in silico study. *Trends Pharmacol. Sci.* 39 229–231. 10.1016/j.tips.2017.12.002 29295742

[B79] Zuo H. Zhang Q. Su S. Chen Q. Yang F. Hu Y. (2018). A network pharmacology-based approach to analyse potential targets of traditional herbal formulas: an example of Yu Ping Feng decoction. *Sci. Rep.* 8:11418. 10.1038/s41598-018-29764-1 30061691 PMC6065326

